# Genetic structure and relationships within and between cultivated and wild korarima [*Aframomum corrorima* (Braun) P.C.M. Jansen] in Ethiopia as revealed by simple sequence repeat (SSR) markers

**DOI:** 10.1186/s12863-017-0540-4

**Published:** 2017-08-01

**Authors:** Dagmawit Chombe, Endashaw Bekele, Tomas Bryngelsson, Abel Teshome, Mulatu Geleta

**Affiliations:** 1 Department of Microbial, Cellular and Molecular Biology, Addis Ababa University, P. O. Box, 1176 Addis Ababa, Ethiopia; 2 Department of Plant Breeding, Swedish University of Agricultural Sciences, P.O. Box 101, -23053 Alnarp, SE Sweden

**Keywords:** *Aframomum corrorima*, Korarima, Genetic diversity, Simple sequence repeats, Genetic structure, Genetic differentiation, Gene flow

## Abstract

**Background:**

Korarima [*Aframomum corrorima* (Braun) P.C.M. Jansen] is a spice crop native to Ethiopia. Understanding the extent and partitioning of diversity within and among crop landraces and their wild relatives is among the first steps in conserving and measuring their genetic potential. The present study is aimed at characterizing the population genetic structure and relationships between cultivated and wild korarima in the southwestern part of Ethiopia.

**Results:**

We analyzed a total of 195 individuals representing seven wild and fourteen cultivated populations. Eleven polymorphic simple sequence repeat (SSR) markers were used. We observed a total of 53 alleles across the eleven loci and individuals. In total, 32 alleles were detected in the cultivated populations, whereas 49 alleles were detected in the wild populations. We found higher genetic diversity in wild populations than in the cultivated counterpart. This result implies the potential of wild korarima as a possible source for novel alleles contributing to the improvement of cultivated korarima. Analysis of molecular variance (AMOVA) showed significant but low differentiation between cultivated and wild korarima populations. Similarly, neighbour-joining and STRUCTURE analyses did not group cultivated and wild populations into two distinct clusters. The lack of clear differentiation between cultivated and wild populations could be explained by historical and contemporary gene flow between the two gene pools.

**Conclusion:**

The 11 SSR loci developed in this study could be employed to examine genetic diversity and population structure of korarima in other countries as well as other *Aframomum* species. From the five administrative zones considered in this study, the Bench-Magi and Sheka zone showed populations with high genetic diversity, and these populations could be used as a potential starting point for in-situ and ex-situ germplasm conservation and korarima improvement through breeding programs after proper agronomic evaluation.

**Electronic supplementary material:**

The online version of this article (doi:10.1186/s12863-017-0540-4) contains supplementary material, which is available to authorized users.

## Background

Korarima (*Aframomum corrorima* (Braun) P.C.M. Jansen) belongs to the monocotyledonous family Zingiberaceae [1]. It is an herbaceous, perennial and aromatic species *native to Ethiopia* [2, 3]. Korarima grows usually with strong fibrous subterranean scaly rhizomes and leafy stems reaching 1–2 m in length. The position of stigma in the flower is below or against the base of the thecae of the anther. Although it is usually self-pollinated, occasional cross-pollination by insects is possible due to the presence of large nectaries at the top of the ovaries [4].

Korarima, also called “false cardamom”, is a part of daily Ethiopian dishes as it is used for preparation of curry powder for culinary purposes. The seeds are used to flavour coffee, special kinds of bread, butter and all kinds of sauces [3]. From a survey by [5], korarima seeds, pods, leaves, rhizomes and flowers are all used in Southern Ethiopia as traditional medicine for different kinds of human and animal ailments. Korarima also plays a role in soil conservation as the rhizomes and leaves spread on the ground covering and protecting the soil from erosion in hilly areas year-around [5]. There is also a demand for korarima in the neighboring countries to Ethiopia as well as in Arabia and Europe where it has long been highly prized as a spice [6]. Therefore, this spice could be developed into an important commodity if necessary attention is given to its research and genetic improvement. The beneficial characteristics of the species can be further enhanced through plant breeding. However, data on genetic characterization of this species is scarce and hence considered necessary before any breeding work can commence. Determination of genetic diversity and population structure are prerequisites of breeding programs and a first step in the development and evaluation of plant genotypes. Phenotypic traits may not give reliable estimates of genetic diversity as these traits are influenced by environmental factors and are limited in number [7]. On the other hand, genetic diversity based on molecular data can potentially facilitate conservation and can be employed as a tool for mining germplasm collections for genomic regions associated with adaptive or agronomically important traits [8]. Microsatellites or simple sequences repeats (SSRs) are tandemly repeated motifs of two to six nucleotides found in all prokaryotic and eukaryotic genomes [9]. Microsatellites are co-dominantly inherited, highly abundant, polymorphic, multi-allelic and reproducible. Hence they have become one of the most desirable molecular markers for use in genetic studies [10]. Interestingly, SSR has been the marker of choice for assessment of genetic diversity in many plant species such as field pea [11], *Sorghum bicolor* [12], Arabica Coffee [13] and chickpea [14]. SSR markers were also used for analysis of phylogenetic relationships [15], marker assisted selection [16], construction of genetic linkage maps [17] and quantitative trait loci maps [18].

The genus *Aframomum* lacks molecular markers that can be used for assessment of its genetic diversity and for other applications. It was, therefore, essential to develop new molecular markers such as microsatellites for *A. corrorima*. Microsatellites can be identified by screening DNA sequence databases for target or closely related species. In the present study, eleven new SSR markers were developed and utilized (1) to evaluate the genetic variation of wild and cultivated korarima populations in southwestern Ethiopia (2) to determine the presence of indirect evidence for crop-wild hybridization by testing for admixture of SSR alleles in cultivated and wild korarima populations from the same regions (3) and to determine potential factors shaping the population genetic structure of cultivated and wild korarima in Ethiopia.

## Methods

### Plant material

**Fig. 1:**
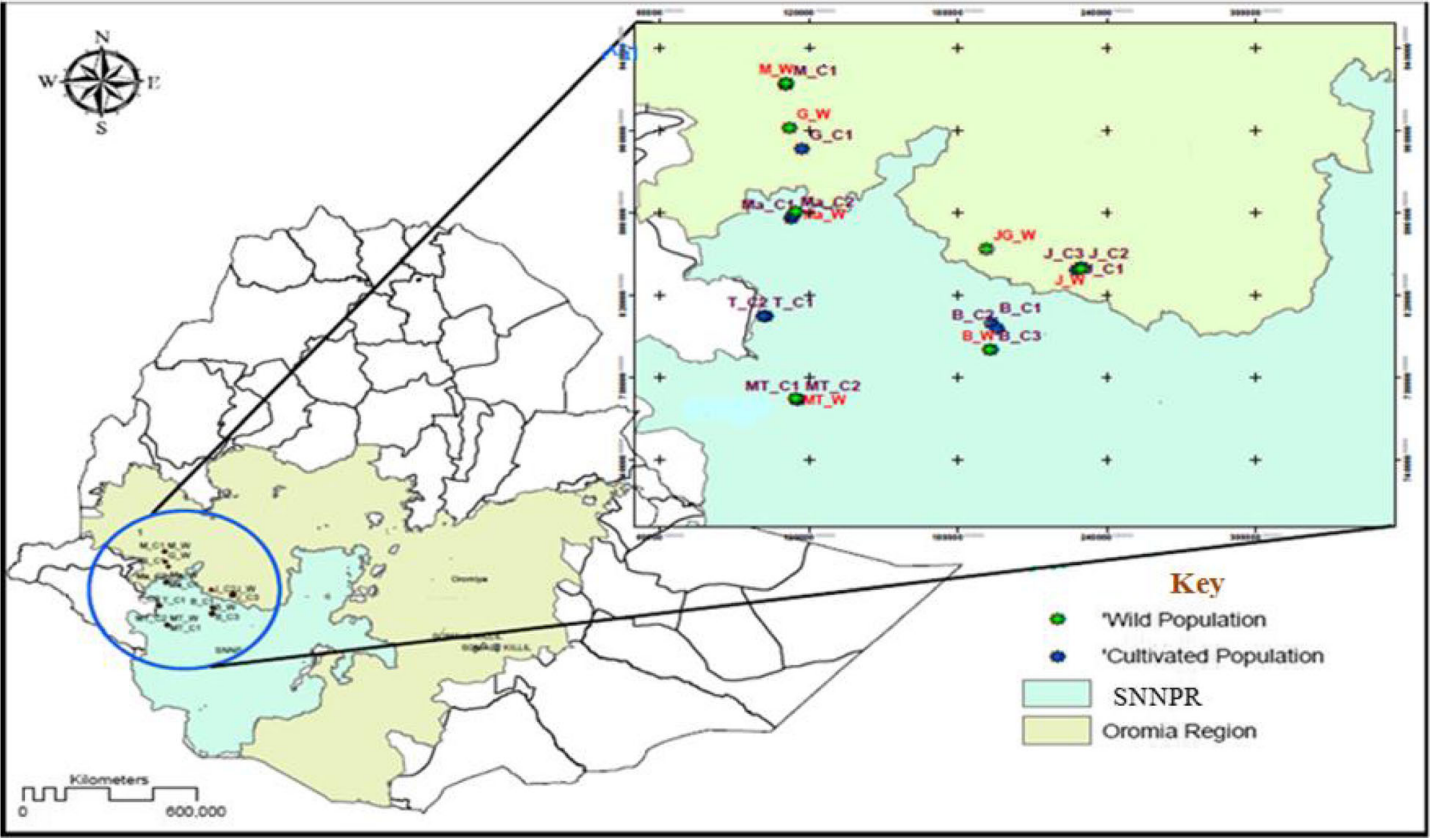
Regional map of Ethiopia showing the wild and cultivated korarima collection sites, SNNPR in the key corresponds to Southern Nations, Nationalities, and People’s Region. The map was constructed based on geographic coordinates and elevation data gathered from each collection sites using global positioning system (GPS)

For this study, 195 individuals from 21 korarima populations (14 cultivated and 7 wild) were sampled across five zones/areas (Illubabour, Jimma, Sheka, Kefa and Bench-Maji) in the southwestern part of Ethiopia (Fig 1). Cultivated populations include korarima samples collected directly from the farmers filed and the wild populations were sampled from the forest. Formal identification of the samples was undertaken based on the description listed on flora of Ethiopia and Eritrea [19]. To collect korarima plant samples from the wild and farmers filed, permission was obtained from the local managers and the farmers respectively. All populations were represented by 10 individuals except three (Mizan-Teferi_C1, Masha_C1 and Metu_W) which were represented by 9, 3 and 3 individuals, respectively (Additional file 1). The distance between sampled plants within each population was at least 20 m. This was done in order to increase the likelihood of representing the genetic variation of each population with limited number of individual plants. Two young leaves were collected from each plant and sealed within plastic bags containing silica gel for DNA extraction. The samples were taken to genetics research laboratory at Addis Ababa University, Ethiopia, and stored at room temperature until the DNA extraction was conducted. Standard material transfer agreement to Sweden was obtained from Ethiopian Institute of Biodiversity conservation (IBC). A copy of each sample gathered was deposited in IBC gene bank for future utilization.

### DNA extraction

Total genomic DNA was isolated from about 1 gram of pulverized leaf sample following a modified CTAB method employing triple extractions to yield optimal amounts of high quality DNA [20]. Genomic DNA from the second extractions was used for PCR amplification, as it was high in both quality and quantity when analyzed using a Nano Drop^®^ ND-1000 spectrophotometer (Saveen Werner, Sweden). QIAamp® 96 DNA QIAcube® HT Kit was used to extract DNA from samples that we failed to isolate in desired quality and quantity using the CTAB method.

### DNA database searches and development of genomic-SSR markers

Since there are no microsatellite containing DNA sequences of *Aframomum* in the NCBI nucleotide database, we used DNA sequences of *Alpinia* in GenBank to develop genomic SSR markers for *Aframomum*. *Alpinia* was used as it is the most closely related genus to *Aframomum* within the Zingiberaceae family [21] which has genomic resources at NCBI. We used 1498 DNA sequences of various *Alpinia* species in NCBI for mining SSRs. DNA sequences that contain SSR with two to six nucleotide repeat motif were identified using WebSat web-based software [22]*.* The analysis revealed that only 1.5% of these sequences contain SSRs and they all were from *Alpinia oxyphylla*. After evaluating the suitability of these sequences for designing primers, 23 SSR primer-pairs were designed using the Primer3 primer designing program [23].

### SSR PCR amplification

The 23 SSR primer-pairs were initially tested for successful amplification of target site and capacity to detect polymorphism among genotypes (Additional file 2). Among these primers, 12 failed to amplify the target loci in *Aframomum corrorima* cultivated and wild populations. The remaining 11 primer-pairs successfully amplified their target loci in *A. corrorima* populations and hence used for genetic diversity analysis (Table 1). The forward primers of these primer-pairs were labeled at the 5^′^-end with either 6-FAM™ or HEX™ fluorescent dyes. In order to avert Taq DNA polymerase tendency to add non templated nucleotide to the PCR product as described in Ballard et al. [24], the reverse primers were *PIG*-tailed with “*GCTTCT*”.

**Table 1:** List of primer-pairs developed and used to amplify the SSR loci in this study

Locus	SSAN	Primer sequence (5^′^-3^′^)		Repeat motifs	Expected size	Observed repeat Motifs	Allele size range^a^
*Afco*_2	JX422069.1	F	TTGACTTGGGTATGGCAAAA	(AG)_13_	230	-	212–242
		R	AAGGTCGAGCAGGAGTAGCA				
*Afco*_3	JX422068.1	F	GAATTCATGTTCTTGAGAAAAGTTTG	(AG)_7_	198	(GA)_2_	191–200
		R	GCCAAATGAACGGACAGATT				
*Afco*_5	JX422066.1	F	TGACTCCAAACTTGCAGGAG	(CT)_8_	160	(CT)_12_	160–171
		R	AGCAGATCAATGCACGTGAG				
*Afco*_6	JX422065.1	F	TCGACATGAAATCCCTACGAGA	(AG)_15_	243	(AG)_12_	230–253
		R	GAGCTGTGAAGTGAAAGGGC				
*Afco*_8	JX422063.1	F	GCTAACTTGTCTTTCCTATTTCTCC	(CT)_13_	239	*	229–245
		R	TGGAAGCTGCATTCACTGAG				
*Afco*_11	JX422060.1	F	AATGCTTCTAGCTGGTTCGAC	(GT)_7_	241	(GT)_6_	240–260
		R	CCTTGAATTTTATATTTCTTCCAGATG				
*Afco*_14	JX422057.1	F	CCTTCCACGGTGTCTCATTT	(GA)_19_	281	(GA)_10_	280–285
		R	TCATCCAAAACTTCAATCATGG				
*Afco*_15	JX422056.1	F	ATCGATGGGATCGCCTTAC	(GA)_19_	292	(GA)_9_	256–262
		R	GACGTCACGAATGTTGGTTG				
*Afco*_19	JX422052.1	F	CAGACGAGAGGAGGGAGATG	(GA)_17_	373	(GA)_9_	356–364
		R	CTCTGTGAGCCGTTCAATCC				
*Afco*_21	JX422050.1	F	CGACAAGGAGGAGAAGAGGT	(GA)_14_	250	(GA)_6_ GAAAGG (GA)_7_	250–260
		R	CCAACAGCCCTTCTTTTTGA				
*Afco*_22	JX422049.1	F	GAAGAAGCGTTGGTGAGAGG	(TC)_20_	468	(TC)_9_	449–455
		R	CTGTGTCGTCCAGCCGTATT				

^a^= refers to allele size across all individuals included in the study; *SSAN* source sequence accession number; *= The SSR are located in the trimmed (low quality) part of the sequence and hence the length of the repeat motif could not be determined; − = sequencing failed

A total volume of 25 μl containing 25 ng genomic DNA, 1 *×* PCR buffer (10 mM Tris-HCl, pH 8.3 and 50 mM KCl), 0.3 mM dNTPs, 0.3 μM forward and reverse primers, 1.5 mM MgCl_2_ and 1 U Dream *Taq* DNA polymerase (Sigma, Germany) was used for PCR reactions. The reactions were performed using a GeneAMP PCR system 9700 thermo cycler (Applied Biosystems Inc. USA) in 96-well plates. PCR amplification reaction consisted of 3 min preliminary denaturation at 95 °C, 30 s touchdown denaturation at 94 °C for nine cycle, 30 s annealing at 58 °C (in every cycle annealing temperature was decreased by *−*1 °C) and elongation for 45 s at 72 °C, afterward 29 cycles of denaturation at 94 °C for 30 s, annealing at 48 °C for 30 s, and elongation at 72 °C for 45 s. Following completion of the 29 cycles, a 20 min final elongation at 72 °C was included to allow completion of the reactions. Quality check on the PCR products were conducted with gel electrophoresis and PCR products were stored at 4 °C until further use.

### Gel electrophoresis and genotyping

For each locus, amplification and quality was confirmed by running 7 μl of the PCR products mixed with 2 μl of 6× loading dye on ethidium bromide containing 1.5% agarose gels. A DNA ladder with the size of 50 bp (GeneRuler™ Fermentas Life Sciences) was used as fragment size marker. The PCR products were multiplexed into three panels where markers with different fluorescent dyes and sizes placed together. ABI Prism 3730 DNA Analyzer (Applied Biosystems) was used for subsequent analysis of the multiplexed PCR products at Swedish University of Agricultural Sciences, Alnarp, Sweden.

### Confirmation of SSRs using DNA sequencing and analysis

To confirm that the amplified products are the target microsatellite loci, the PCR products of two individuals of *A. corrorima* (one cultivated and one wild) amplified by the 11 primer-pairs were sequenced. Before the PCR products were sent for sequencing to Eurofins Genomics (Germany), they were purified using the Qiaquick PCR purification kit. Then, each purified PCR product was mixed with corresponding forward primer, which was used as sequencing primer. The final edited sequences were searched in NCBI through Basic Local Alignment Search Tool (BLAST) to find out if it hits with the original SSR containing microsatellite sequence or any other similar sequence in the database. Similar sequences were aligned using Clustal X version 2.1 software [25], and then sequences were edited using BIOEDIT version 7.0.5 [26].

### Data analysis

Allele peak and its size was identified using GeneMarker® V2.2.0 software (Soft Genetics, LLS, State College, Pennsylvania) based on the Genescan-500 LIZ internal size standard. After pre-analysis of peaks using default settings in GeneMarker and visual examination, peaks with threshold intensity of 200 were accepted for scoring.

For estimating observed heterozygosity, expected heterozygosity, gene flow and allele frequency based genetic distance analyses, POPGENE software version 1.31 [27] was used. The estimate of gene flow (Nm) was generated based on equation provided in [28]. GenAlEx 6.5 software V3.25 [29] was used to compute private alleles by population and locus and to calculate pair-wise F_ST_. Analysis of molecular variance (AMOVA) was conducted using Arlequin 3.0 according to Excoffier et al. [30]. A neighbor-joining (NJ) and UPGMA (unweighted pair group method using arithmetic average) dendrogram was constructed based on pair-wise simple matching dissimilarity index using DARwin6 software [31]. In the NJ tree construction a random re sampling of five individuals from 19 population and three individuals from two populations [(5 × 19) + (3 × 2) = 101] were done in order to obtain a better viewing of clusters due to reduced number of samples.

The pattern of population structure and detection of probable introgression was visualized using a Bayesian model based clustering method implemented in STRUCTURE software version 2.2 [32]. The site of collection was assumed as the putative population origin for each individual during analysis. The admixture model with a burn-in period of 10,000 and 100,000 Markov Chain Monte Carlo (MCMC) replications was implemented for gathering data from K = 1 to K = 21 groups. Five independent replicates were run for each K value. In this way the proportion of membership of each individual in each population was estimated as described in Falush et al. [33]. Following the simulation method of Evanno et al. [34], the optimum number of clusters was estimated between K = 1 and K = 21 using the web based software STRUCTURE HARVESTER v0.6.8 [35]. The clusters across the replicates were aligned using CLUMPP software [36] and then DISTRUCT software [37] was used to display population clusters.

## Results

In the present study, a total of 11 genomic-SSR loci was developed. Detailed information of these SSR- loci such as source sequence accession numbers, forward and reverse primers, repeat motifs, expected fragment size and observed fragment size range was described in Table 1. All of these SSR loci have dinucleotide repeat motifs and were amplified in all populations of korarima. At loci *Afco*_8 and *Afco*_14, only one allele was detected within cultivated populations (across the 132 individuals) and within wild populations (across 63 individuals), respectively, whereas the remaining nine loci were polymorphic within both groups of populations. Therefore, only the 10 polymorphic loci within each group were used to compute genetic diversity parameters when cultivated and wild populations were analyzed separately.

**Fig. 2:**
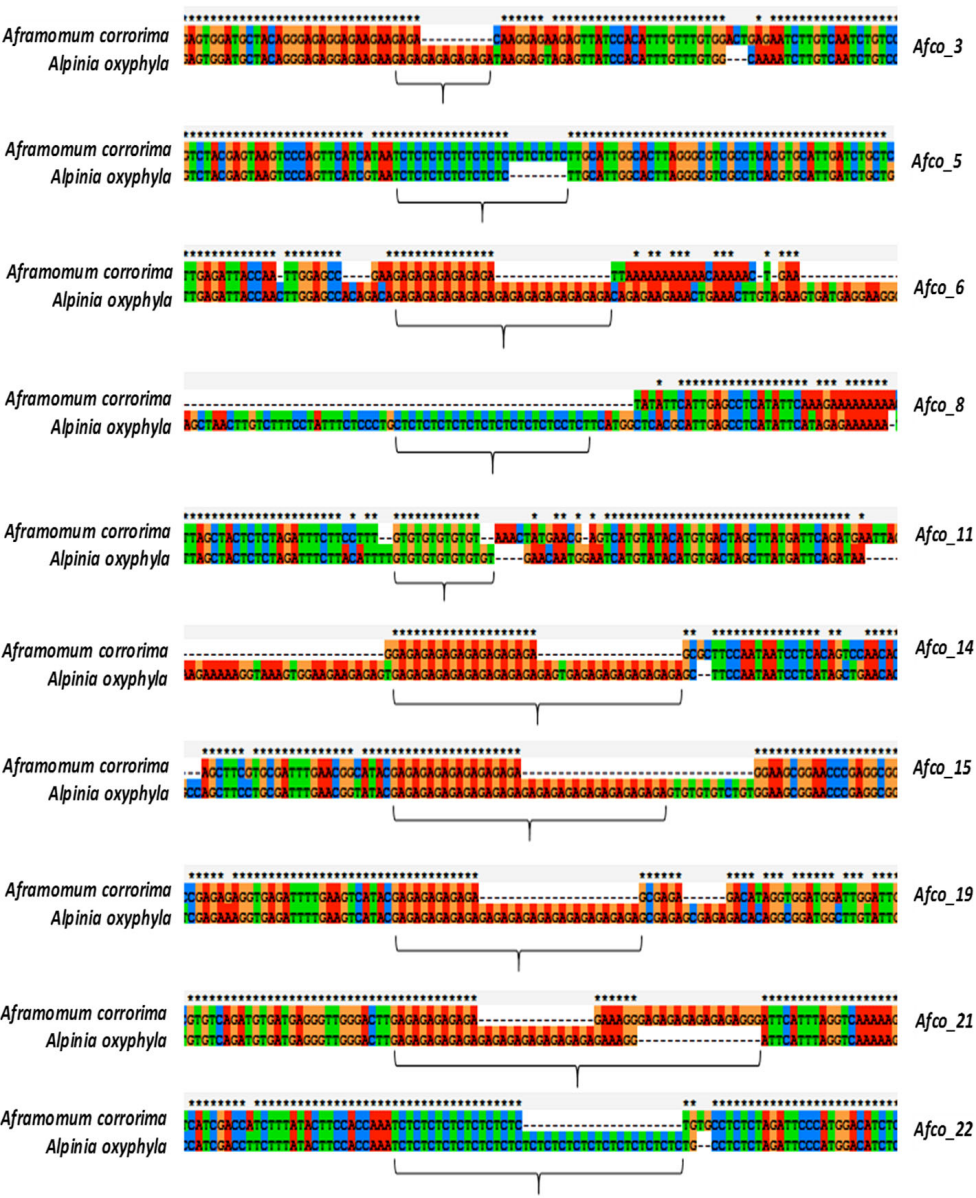
Short segments of the alignment of microsatellite containing sequences of *Aframomum corrorima* and *Alpinia oxyphyla* species for 10 of the 11 SSR loci used in the present study. The locus names are given to the right of each aligned sequences. The SSR region for each locus is shown by curly bracket. Note: sequencing of *Afco*_2 failed and hence is not included here

The sequences of amplified products matched the original *A. oxyphylla* sequences used for designing the SSR primer-pairs except in the case of *Afco*_2. The sequencing of the PCR product amplified by *Afco_2* failed. All sequences that matched the original *A. oxyphylla* sequences contained the target microsatellites except in the case of *Afco*_8 which had poor sequence quality in the microsatellite containing region and hence trimmed off (Fig 2).

**Fig. 3:**
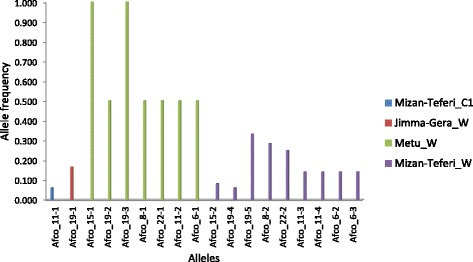
List of private alleles unique to one of the four populations shown on the right and its frequencies at six SSR loci

Loci like *Afco*_6, *Afco_8*, *Afco*_11, *Afco*_15, *Afco*_19 and *Afco*_22 produced private allele(s) that are unique to a single population. In the case of the cultivated populations, only one population (Mizan-Teferi_C1) produced a private allele, which was at locus *Afco*_11 (allele frequency = 6.3%), whereas private alleles were detected in three wild populations (Jimma-Gera_W, Metu_W and Mizan-Teferi_W). The highest number of private alleles (9) was detected in the Mizan-Teferi_W population across six loci but with a low frequency ranging from 6.3% to 33.3% (Fig. 3).

In total across the 10 loci, 32 alleles were detected in the 14 cultivated populations, whereas 49 alleles were detected in the seven wild populations. The observed number of alleles (na) per locus was from two to five in cultivated populations and from four to eight in wild populations. The effective number of alleles (ne) per locus varied from 1.02 (*Afco*-19) to 2.39 (*Afco*_11) for cultivated populations and from 1.42 (*Afco*_8) to 4.87 (*Afco*_2) for wild populations. The Shannon diversity index (I) per locus ranged from 0.06 (*Afco*_19) to1.02 (*Afco*_2 and *Afco*_11) for cultivated populations and from 0.63 (*Afco*_8) to 1.72 (*Afco*_2) for wild populations. The average Shannon diversity index for all loci was 0.76 and 1.06 for cultivated and wild populations, respectively. Observed heterozygosity (Ho) ranged from zero at locus *Afco*-19 to 0.88 at locus *Afco*-11 in cultivated populations and from zero at locus *Afco*-19 to 0.86 at locus *Afco*-11 in wild populations. The average observed heterozygosity across all loci was 0.63 and 0.48 for cultivated and wild populations, respectively. Expected heterozygosity (He) ranged from 0.02 at locus *Afco*-19 to 0.58 at locus *Afco*-11 in cultivated populations and from 0.30 at locus *Afco*-8 to 0.80 at locus *Afco*-2 in wild populations. The average expected heterozygosity for all loci was 0.48 and 0.57 for cultivated and wild populations, respectively (Table 2). The estimate of gene flow (Nm) based on F_ST_ across all populations was 0.94 (Table 2).

**Table 2:** Estimates of different genetic diversity parameters and gene flow for *A. corrorima* at each polymorphic microsatellite loci based on (A) all populations, (B) only cultivated populations, and (C) only wild populations

Locus	Sample size	na	ne	I	Ho	He	Av.Het	Nm
(A) All Populations								
* Afco*_2	270	8	3.16	1.38	0.66	0.69	0.53	0.87
* Afco*_3	322	5	2.42	1.07	0.59	0.59	0.50	1.60
* Afco*-5	284	5	2.58	1.10	0.66	0.61	0.55	1.50
* Afco*-6	272	5	1.94	0.78	0.71	0.49	0.43	2.10
* Afco*-8	284	4	1.09	0.22	0.02	0.08	0.06	0.19
* Afco*-11	300	8	2.45	1.10	0.87	0.59	0.55	3.02
* Afco*-14	58	2	1.62	0.57	0.10	0.39	0.14	0.08
* Afco*-15	274	4	2.09	0.80	0.84	0.52	0.46	1.45
* Afco*-19	228	4	1.13	0.30	0.00	0.12	0.05	0.13
* Afco*-21	296	4	2.64	1.06	0.75	0.62	0.54	1.32
* Afco*-22	260	4	1.91	0.75	0.68	0.48	0.40	1.25
Mean	259	4.82	2.09	0.83	0.54	0.47	0.38	0.94
St. Dev		1.78	0.64	0.36	0.33	0.20	0.20	
(B) Cultivated Populations								
* Afco*-2	188	5	2.28	1.02	0.68	0.56	0.47	1.91
* Afco*-3	224	5	2.27	1.00	0.71	0.56	0.52	3.73
* Afco*-5	196	4	2.17	0.88	0.71	0.54	0.51	3.20
* Afco*-6	208	2	1.91	0.67	0.76	0.48	0.47	9.48
* Afco*-11	228	5	2.39	1.02	0.88	0.58	0.55	6.54
* Afco*-14	38	2	1.92	0.67	0.16	0.49	0.21	0.14
* Afco*-15	208	2	1.99	0.69	0.87	0.50	0.49	11.36
* Afco*-19	170	2	1.02	0.06	0.00	0.02	0.02	1.62
* Afco*-21	218	3	2.33	0.92	0.79	0.57	0.53	3.80
* Afco*-22	210	2	1.87	0.66	0.73	0.47	0.46	9.63
Mean	189	3.2	2.01	0.76	0.63	0.48	0.42	1.94
St. Dev		1.40	0.39	0.29	0.29	0.17	0.17	
(C) Wild Populations								
* Afco*-2	82	8	4.87	1.72	0.61	0.80	0.67	1.21
* Afco*-3	98	4	2.24	0.95	0.31	0.56	0.47	1.68
* Afco*-5	88	5	3.40	1.34	0.55	0.71	0.62	1.28
* Afco*-6	64	5	1.93	0.94	0.56	0.49	0.36	1.18
* Afco*-8	72	4	1.42	0.63	0.08	0.30	0.18	0.23
* Afco*-11	72	7	2.64	1.21	0.86	0.63	0.54	1.53
* Afco*-15	66	4	2.40	0.99	0.76	0.59	0.42	0.54
* Afco*-19	58	4	1.55	0.72	0.00	0.36	0.13	0.12
* Afco*-21	78	4	3.29	1.24	0.64	0.71	0.57	0.97
* Afco*-22	50	4	1.96	0.92	0.44	0.50	0.29	0.38
Mean	73	4.90	2.57	1.06	0.48	0.57	0.42	0.74
St. Dev		1.44	1.04	0.32	0.28	0.15	0.18	

Note: *Afco-8* and *Afco-14* were monomorphic within cultivated and wild populations, respectively, and hence were not included when the data for the cultivated and wild populations were analyzed separately. *na* observed number of alleles, *ne* effective number of alleles, *I* Shannon information index, *Ho* observed heterozygosity, *He* expected heterozygosity, *Av. He* average heterozygosity, *Nm* gene flow estimated from Fst = 0.25(1 - Fst)/Fst

Percentage of polymorphic loci (PPL), observed and expected heterozygosity, Shannon diversity index was determined for each population. The maximum PPL per population was 100%, which was observed in the Mizan-Teferi_W from the Bench-Maji Zone, while the lowest was 60% for Masha_W (Sheka zone), both of which are wild populations. In the cultivated populations, the highest PPL was 90% which was recorded in seven of the 14 populations (Gore_C1, Jimma_C1, Mizan-Teferi_C1, Masha_C2, Tepi_C1, Tepi_C2 and Bonga_C1) and the least was 70% for the Masha_C1 population (Table 3).

**Table 3:** Percentage of polymorphic loci (PPL), Shannon’s diversity index (I), observed heterozygosity (Ho) and expected heterozygosity (He) for cultivated and wild populations of *Aframomum corrorima*

Zone	Population	PPL	I	Ho	He	Sample size
Cultivated Populations						
Illubabour	Gore_ C1	90	0.75	0.58	0.54	10
	Metu _C1	80	0.71	0.57	0.47	10
	Average	85	0.73	0.58	0.51	
Jimma	Jimma_ C1	90	0.56	0.54	0.43	10
	Jimma_C2	80	0.61	0.69	0.45	10
	Jimma_C3	80	0.56	0.55	0.40	10
	Average	83.33	0.58	0.59	0.43	
Bench-Maji	Mizan-Teferi_C1	90	0.66	0.73	0.49	9
	Mizan-Teferi_C2	80	0.63	0.55	0.43	10
	Average	85	0.65	0.64	0.46	
Sheka	Masha_C1	70	0.52	0.72	0.50	3
	Masha_C2	90	0.82	0.62	0.56	10
	Tepi_C1	90	0.68	0.60	0.47	10
	Tepi_C2	90	0.69	0.50	0.48	10
	Average	85	0.68	0.61	0.50	
Kefa	Bonga_C1	90	0.66	0.78	0.53	10
	Bonga_C2	80	0.66	0.65	0.47	10
	Bonga_C3	80	0.58	0.74	0.43	10
	Average	83.33	0.68	0.72	0.48	
Wild Populations						
Illubabour	Metu_W	80	0.56	0.73	0.48	3
	Gumero_W	80	0.65	0.51	0.43	10
	Average	80	0.61	0.62	0.46	
Jimma	Jimma-Gera_W	90	0.73	0.38	0.50	10
	Jimma_W	80	0.56	0.42	0.43	10
	Average	85	0.65	0.40	0.47	
Bench-Maji	Mizan-Teferi_W	100	1.03	0.52	0.64	10
Sheka	Masha_W	60	0.52	0.30	0.38	10
Kefa	Bonga_W	80	0.71	0.45	0.46	10

Among the wild populations, the highest Shannon diversity index was recorded for Mizan-Teferi_W (*I* = 1.03) and the lowest for Masha_W (*I* = 0.52), whereas in the cultivated populations, the highest was for Masha_C2 (*I* = 0.82) and the lowest was for Masha_C1 (*I* = 0.52). The highest and lowest observed heterozygosity was scored for Bonga_C1 (Ho = 0.78) and Tepi_C2 (Ho = 0.50) in the cultivated populations, and Metu_W (Ho = 0.73) and Masha_W (Ho = 0.30) in the wild populations. The expected heterozygosity in Masha_C2 (He = 0.56) was the highest and the lowest was 0.40 (for Jimma_C3) among the cultivated populations. Among the wild populations, the highest and the lowest expected heterozygosity was recorded in Mizan-Teferi_W (He = 0.64) and Masha_W (He = 0.38), respectively (Table 3).

Two complementary approaches were used to explore the genetic diversity structure: analysis of molecular variance (AMOVA) and pair-wise fixation index (F_ST_). In the case of AMOVA, the total genetic variation was partitioned into three hierarchical levels: among groups (cultivated vs wild), within groups among populations and within populations. Pair-wise F_ST_ was estimated for all populations as well as for cultivated and wild populations, separately.

AMOVA revealed highly significant (*P* < 0.001) but low level of genetic differentiation (F_ST_ = 0.04) between the wild and cultivated populations. Of the total genetic variation, 5.7% was attributable to the variation between cultivated and wild korarima. The analysis revealed absence of differentiation among the cultivated populations as well as wild populations (Table 4).

**Table 4:** Analysis of molecular variance (AMOVA) for the cultivated and wild korarima populations

Source of variation	d.f.	Sum of squares	Variance components	%age of variation	*P-value*
AG	1	13.555	0.08193 V_a_	5.74	Vc and *F*_*ST*_ = 0.000
APWG	19	16.850	−0.02892 V_b_	−2.03	Vb and *F*_*SC*_ = 0.069
WP	335	460.267	1.37393 V_c_	96.29	Va and *F*_*CT*_ = 0.000
Total	355	490.671	1.42694		
Fixation Index	F_ST_ = 0.04				

*AG* among groups of populations (cultivated vs wild), *APWG* among populations within groups (cultivated and wild), *WP* within populations

Both cultivated and wild populations were further divided into smaller groups separately and AMOVA analysis was conducted. First, both cultivated and wild populations were grouped into two: Oromia and SNNP based on their region of origin. Then, the populations where grouped into five groups based on their zones of origin. In cultivated korarima, no genetic differentiation was obtained among populations, regions and zones (F_ST_ = −0.05; *P* < 0.001). However, in the case of wild populations significant differentiation was obtained at population (F_ST_ = 0.07; *P* < 0.001), region (F_ST_ = 0.06; *P* < 0.001) and zone (F_ST_ = 0.09; *P* < 0.001) levels (Table 5).

**Table 5:** F_ST_ based estimates of genetic differentiation of cultivated and wild korarima populations at various levels

Populations	Differentiation at	Mean F_ST_	*P*-value
Cultivated	Population level	−0.05	0.000
	Region level	−0.05	0.000
	Zone level	−0.05	0.000
Wild	Population level	0.07	0.000
	Region level	0.06	0.000
	Zone level	0.09	0.000

The Nei’s standard genetic distance between cultivated and wild korarima populations ranged from 0.06 (T_C1vs G_W) to 1.56 (B_C1 vs M_W). The genetic distance within cultivated populations ranged from 0.01 (MT_C2 vs M_C1) to 0.19 (B_C3 vs M_C1). The genetic distance within wild populations ranged from 0.04 (Ma_W vs B_W and Ma_W vs JG_W) to 1.64 (M_W vs Ma_W) (Table 6). The Metu wild population (M_W) is distantly related to the rest of the wild populations and cultivated korarima with an observed genetic distance of greater than 1.30 (Table 6).

**Table 6:**
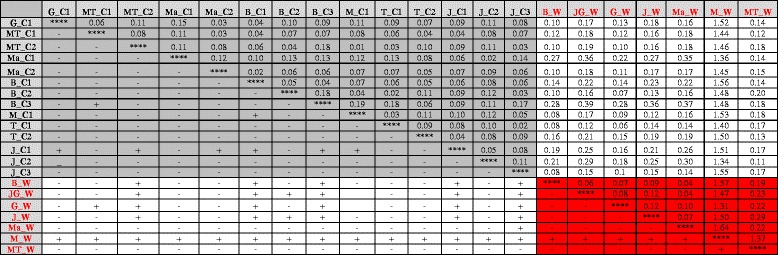
Nei’s standard genetic distance (above diagonal) and pair wise F_ST_ significance (below diagonal) between the 21 korarima populations

+: Significant differentiation among the joint populations (*P* < 0.05)

-: No significant differentiation among the joint populations (*P* > 0.05)

Area shaded with gray color represents pair-wise comparison b/n cultivated population; the one shaded with red color represents pair-wise comparison b/n wild populations. The unshaded region is b/n cultivated and wild population. G_C1 = Gore_C1; MT_C1 = Mizan-Teferi_C1; MT-C2 = Mizan-Teferi_C2; Ma_C1 = Masha_C1; Ma_C2 = Masha_C2; B_C1 = Bonga_C1; B_C2 = Bonga_C2; B_C3 = Bonga_C3; M_C1 = Metu_C1; T_C1 = Tepi_C1; T_C2 = Tepi_C2; J_C1 = Jimma_C1; J_C2 = Jimma_C2; J_C3 = Jimma_C3; B_W = Bonga_W; Jg_W = Jimma-Gera_W; G_W = Gumero_W; J_W = Jimma_W; Ma_W = Masha_W; M_W = Metu_W; MT_W = Mizan-Teferi_W

The comparison of the extent of genetic differentiation within and between cultivated and wild korarima populations (pair-wise F_ST_) is presented in Table 6. Among cultivated korarima, the most differentiated population is J_C1 which is significantly differentiated from six cultivated populations (G_C1, MT_C1, Ma_C2, B_C1, B_C3 and M_C1). Populations B_C1 and B_C3 were significantly differentiated from two cultivated populations (M_C1 and J_C1). Among wild korarima, the most differentiated population was Metu wild (M_W) population. Pair-wise F_ST_ showed that M_W was significantly differentiated from all other wild populations as well as from all cultivated populations.

**Fig. 4:**
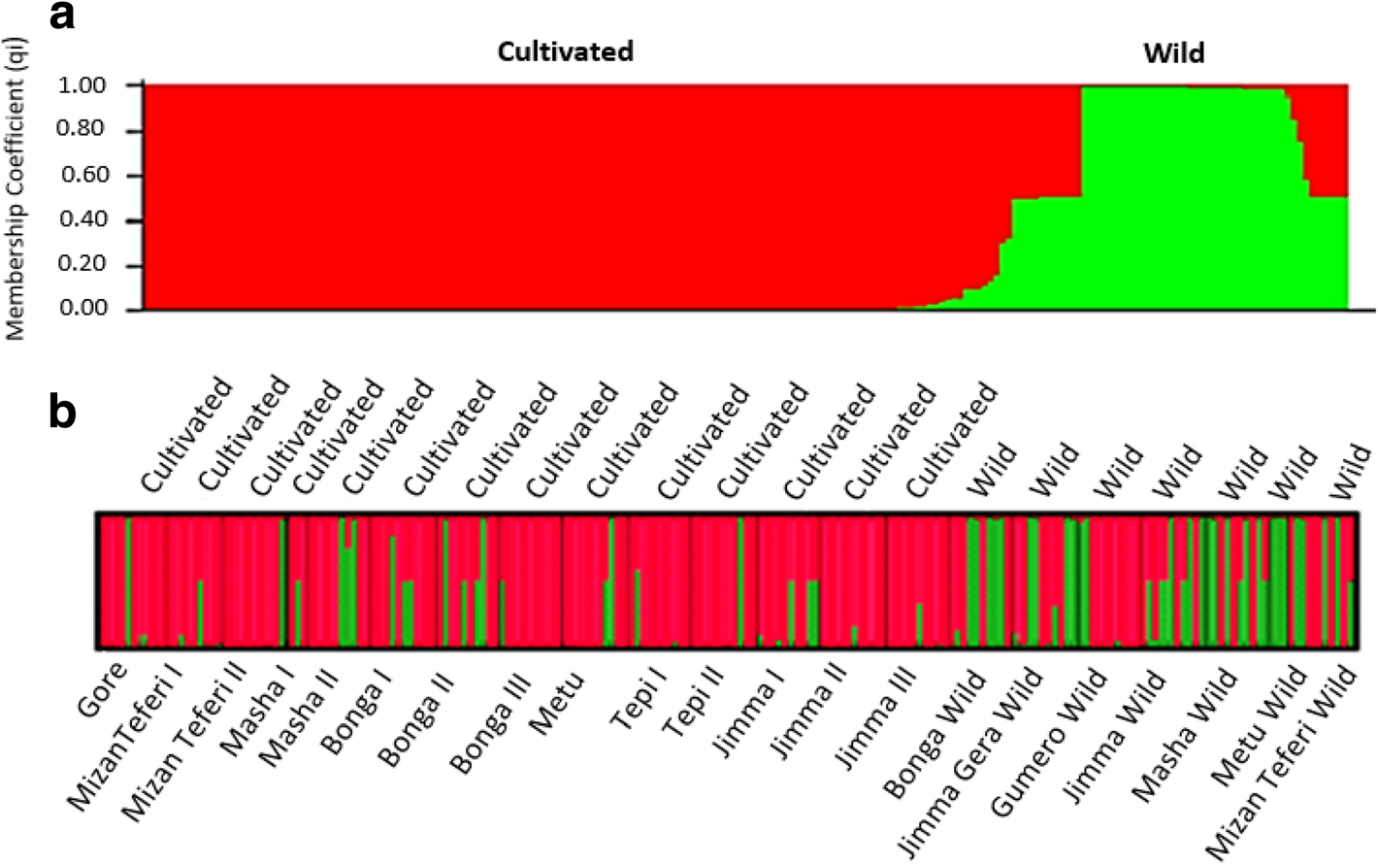
**a** Estimated population structure at K = 2 for the 195 individuals ordered by type and membership fraction (qi). **b** STRUCTURE bar graphs of the entire korarima samples in 21 pre-determined populations (x-axis) at K = 2. The text on top of the figure represents korarima types; the text beneath the figure represents the different populations. Same colors in different individuals indicate that they belong to the same cluster. Different colors for the same individual indicate the percentage of the alleles that placed it in each cluster

Analysis using STRUCTURE HARVERSTER revealed that the maximum peak is at K = 2 (Additional file 3), suggesting that two populations can integrate all individuals included in the present study with the highest likelihood. A graphic representation of estimated membership coefficients of each individual at K = 2 was shown in Fig. 4a and b. Each color represents the proportion of membership of each individual, represented by a vertical line, to the two clusters. Bayesian model-based cluster analysis at K = 2 did not produce distinct differentiation among cultivated and wild individuals (Fig. 4a). In this analysis, we can observe some individuals from both cultivated and wild population jointly assigned to two clusters, following the assumption that an individual was only exclusively assigned to a particular genetic cluster if at least 85% of its genome (i.e. qi ≥0.85) is found in it. In Fig. 4b there is no distinct structure among populations which shows an admixture of populations.

## Discussion

### Genomic-SSR markers

Molecular markers have been widely used in studying genetic diversity and relationships, screening candidate genes associated with the target traits and marker assisted breeding. Microsatellite markers derived from genomic DNA regions have been chosen in many studies for their high levels of allelic variability compared to microsatellites derived from expressed sequence tags (EST-SSRs) [38]. Primer pairs intended on the base of sequence of one species possibly will be used to develop SSR markers for other related species [39]. We used publicly available microsatellite containing genomic sequences of *Alpinia oxyphylla* to develop 11 new SSRs for *Aframomum corrorima*. All of these microsatellites are dinucleotide repeats, which are the most common types of SSRs developed based on genomic DNA sequences [9]. All of these loci were polymorphic when the whole populations studied were considered. However, the number of polymorphic loci was reduced by one when the loci were considered either for cultivated or wild populations. The high rate of marker transferability obtained in the present study suggest that the flanking regions of these loci are sufficiently conserved, and allows comparative analyses of genetic diversity in the genus Aframomum and related genera using these markers. The sequence alignment analysis showed that there was variation in the repeat length of the dinucleotide motifs between the two species *A. corrorima* and *Alpinia oxyphylla* (Fig. 2), which suggests a clear differentiation of these two species at these SSR loci. However, the sequences that cover the SSR containing part of the PCR products were not obtained for *Afco*_2 and *Afco*_8, as sequencing failed for *Afco_2*, and the SSR containing part of *Afco_8* had poor sequence quality, which is attributed to the fact that the SSR is located close to the left primer annealing site.

### Genetic diversity

Nearly half of the loci developed for the diversity study amplified alleles exclusive to a single population (private alleles). The mainly self-pollinating nature of korarima [4] could be one of the main reasons behind the recognition of private alleles in the majority of the loci studied smoothing the possibility of new mutations to remain in the original population due to reduced gene-flow among populations. Among cultivated populations, only one (MizanTeferi_C1) produced a private allele (frequency = 6.25%; Fig. 3). Differences in agricultural practices in order to strengthen their adaptation to local agro-ecological conditions may have contributed to the genetic distinctiveness of this population. Most of the private alleles were found in three wild populations: Jimma Gera_W, Metu_W and Mizan-Teferi_W having 1, 7 and 9 private alleles, respectively (Fig. 3). The presences of these private alleles have important implications because they may be in linkage disequilibrium with genes coding for desirable traits. Thus they could be useful in indicating a particular type of genotype for breeding purposes [40].

When genetic analysis was done for all populations, a total of 53 alleles, which averaged to 4.82 alleles per locus, were detected across 11 loci. The average number of alleles per locus observed in the current study is analogous to a 4.3 alleles/locus that was reported for ginger (*Zingiber officinale*) accessions genotyped with eight SSR markers [41]. However, it is lower than 5.7 alleles/locus reported for *Curcuma longa* [42] and 5.8 alleles/locus for *Piper nigrum* [43] based on data from eighteen and nine microsatellite loci, respectively. The relatively lower number observed in *A. corrorima* and *Z. officinale* could be attributed to their diploid chromosome number, as *C. longa* and *P. nigrum* are triploid and tetraploid, respectively*.* In the present study, *Afco*-2 and *Afco*-11 were the most polymorphic loci both containing eight alleles. Comparable number of alleles per locus was reported in *Z. officinale* (seven alleles; [41]), *C. longa* (nine alleles; [42]) and *P. nigrum* (ten alleles; [43]). Based on 23 polymorphic microsatellites, Zou et al. [44] reported up to 13 alleles per locus in *Alpinia oxyphylla* which is higher than that obtained in the present study.

The average observed heterozygosity (H_O_) obtained in this study was 0.54 (Table 2). This result is lower than that reported in *P. nigrum* (0.62, [43]) and in *A. oxyphylla,* (0.64, [44]). Likewise, the expected heterozygosity (He) reported in this study was lower than that reported in *P. nigrum* (0.72; [43]) and in *A. oxyphylla* (0.62; [44]). The relatively lower genetic diversity observed in this study can be partly explained by combinations of fewer populations and loci studied.

Our study showed higher levels of diversity in wild populations compared to cultivated populations in terms of average number of alleles/locus, Shannon diversity index and expected heterozygosity. The average number of alleles/locus and Shannon diversity index of wild korarima were higher than that of cultivated korarima, 4.9 vs. 3.2 and 1.06 vs 0.76, respectively. The expected heterozygosity (He) showed the same trend with 0.57 and 0.48 for wild and cultivated korarima, respectively (Table 3). The high genetic diversity recorded in the wild populations may suggest the possibility of finding novel alleles contributing to the improvement of cultivated korarima and has to be given special attention for conservation and utilization.

Among wild populations, the highest Shannon diversity index, the highest percentage of polymorphic loci and the highest expected heterozygosity was recorded in Mizan-Teferi_W, a population from the Bench-Maji zone and lowest in Masha_W, a population from the Sheka Zone. The genetic diversity of most cultivated populations is relatively high with all of the populations having a percentage of polymorphic loci higher than or equal to 70% and a Shannon diversity index of more than 0.50. High genetic diversity was observed in Masha_C2 population (Sheka zone) possessing high percentage of polymorphic loci and high Shannon diversity index compared to other cultivated populations. This may indicate that farmers in this area grow a mixture of landraces of korarima or have their own unique culture of conservation. The lowest diversity found in the other two Masha populations (Masha_C1 and Masha_W) in the Sheka zone, which are cultivated and wild in that order, was rather unexpected. The decreased genetic diversity in cultivated Masha_C1 could be due to strong genetic drift which results in a rapid decline of population size or sampling error during individual collection. However, the reduced diversity observed in Masha_W is most likely due to human interference such as digging the rhizomes for planting and other traditional practices. These will result a decline in population size. There is a real risk that if the trend towards a decline in population size is left unchecked, genetic consequences associated with small isolated populations, including genetic drift and inbreeding, will reduce most of the genetic diversity in the Masha wild population. Thus, there is an urgent need to assess the distribution and size of *A. corrorima* populations in this zone and to adopt appropriate *insitu* conservation strategies and collect germplasm for *exsitu* conservation.

### Genetic structure and gene flow

Our analysis did not show distinct divergence between wild and cultivated korarima. Based on the AMOVA analyses, we found a significant but a relatively low level genetic differentiation among populations of cultivated and wild korarima (F_ST_ = 0.04; *P* < 0.001). Pair-wise F_ST_ also showed the presence of low level of crop-wild genetic divergence with each wild population showing a significant differentiation from only less than half of the cultivated population. Several factors can be included in attempts to account for the observed genetic structure in cultivated and wild korarima. The present-day cultivated *A. corrorima* may have been directly introduced to cultivation from wild progenitors inhabiting the same localities and thus may have maintained most of their initial genetic diversity. In Southwest Ethiopia, the cultivation area of *A. corrorima* overlaps with the distribution of natural habitats of wild populations. Because of the sympatric distribution pattern of the wild and cultivated populations, migration from wild to cultivated populations by natural or artificial means may be an ongoing process. Therefore, subsequent gene flow from wild ancestors into cultivated populations (Nm = 0.94; Table 2) may also contribute to the maintenance of a relatively high genetic diversity in cultivated *A. corrorima.*

Crop cultivation impacts not only the amount of genetic variation contained in cultivated populations but also the structure of this variation [45]. Hamrick and Godt [46] reported that the mean value of genetic differentiation among populations of crop species is higher than that of non-crop species. However, our results run contrary to this report. The cultivated (crop) *A. corrorima* populations did not show genetic differentiation among populations (*F*_ST_ = −0.05; *P* < 0.001) whereas wild (non-crop) populations showed significant population differentiation (*F*_ST_ = 0.07, *P* < 0.001). The absence of clear differentiation among cultivated populations can be explained by the movement of genetic material between growers within and among the major production areas, which result in a higher degree of gene flow among cultivated populations (Nm = 1.94, Table 2) and diminish their genetic differences. For wild populations, dispersal of seeds and natural migration between populations is limited, especially when habitats are isolated by geographic barriers such as mountains, rivers or distance. As a result, wild populations are expected to display a relatively greater proportion of variance among populations.

The main evolutionary forces that shape genetic structure in plant populations are gene flow, selection associated with environmental heterogeneity and/or farmer preferences and random genetic drift [47]. For cultivated korarima, there is no differentiation among populations both at the level of regions and zones. The absence of genetic divergence among regions and zones could be attributed to geographical proximity of the regions and zones where korarima is currently cultivated, resulting in high gene flow in the form of exchanges of germplasm among farmers. The overall population differentiation among regions and among administrative zones for wild korarima was significant (*P* < 0.0001) with F_ST_ value of 0.06 and 0.09 respectively (Table 5). Natural barriers and geographic distance between regions restricts the level of gene flow among wild populations and should therefore contribute significantly to the contemporary genetic structure. In addition, the moderate level of differentiation among administrative zones is expected because different altitudes within a geographical zone would provide different degrees of selection pressure for adaptation and could accelerate population differentiation rather than gene flow [48].

STRUCTURE analysis following Evanno’s *adhoc* ΔK method suggested that the 21 populations included in the present study most likely form two clusters (K = 2) (Additional file 3). Similar to the result obtained from AMOVA and pair-wise F_ST_ analysis, divergence between the two types of korarima was not clear at *K* = 2. The mixed clustering pattern of individuals of the cultivated and wild populations was observed in the population structure constructed based on STRUCTURE software. All individual genotypes in each population showed partial membership to both clusters (Fig. 4a and b). In addition, the NJ tree revealed two clusters, each comprising individuals from cultivated and wild populations (Additional file 4). Moreover, the UPGMA dendrogram, based on pair-wise simple matching dissimilarity index among the 21 cultivated and wild populations, showed two clusters with two wild and one cultivated populations as outliers (Additional file 5). Isolation from cultivars is generally considered to be beneficial from the standpoint of preserving the integrity and diversity of wild gene pools. The fact that two wild populations grouped very closely with cultivars suggests that there may be ongoing gene flow between them via hybridization and/or cultivars becoming naturalized as free-living populations. Therefore, the overall result obtained in this study suggests that there is gene flow among the two korarima types (cultivated and wild) due to their relative geographical proximity to each other.

## Conclusions

This study provides a first detailed analysis and estimation of genetic diversity in Ethiopian populations of korarima using 11 genomic SSR markers newly developed based on genomic resources of *Alpinia oxyphylla*. These SSR markers were highly polymorphic within Ethiopian *Aframomum corrorima* used in this study and could be employed to examine genetic diversity and population structure of korarima in other countries as well as other *Aframomum* species. Some of the alleles identified across the 11 SSR loci may be linked to important agronomic traits and have to be further evaluated through their use in korarima mapping populations. Overall, Ethiopian korarima needs broader characterization at both molecular and agro-morphological levels in order to use it in future korarima improvement. The data also shows the power of SSR markers to define how korarima genetic diversity is structured in Ethiopia. This can lead to a better management of the populations in-situ and ex-situ and promote the use of these populations in further breeding programs. Among the five zones in southwestern Ethiopia considered in this study, both Bench-Maji and Sheka harbor populations with high genetic variation and could be used as a starting in-situ conservation sites. We expect that more extensive sampling of korarima in other regions of Ethiopia followed by molecular characterization would reveal additional target sites for in-situ conservation, for development of core collection for long-term ex-situ conservation, as well as possible evidence of crop–wild gene flow. The present finding is a timely contribution, considering korarima’s high economic and endogenous value, and current challenges in korarima biodiversity management and conservation.

## Additional files

Additional file 1: Passport data of *A. corrorima* samples collected from different sites in Ethiopia. (DOCX 17 kb)

Additional file 2: Characteristics of 23 microsatellite primers developed for *Aframomum corrorima* (The bold one are used for genetic diversity analysis) (DOCX 16 kb)

Additional file 3: A biplot detected the maximum peak at K = 2 (the optimum number of clusters) based on Evanno et al. 2005 [35] prediction. (PDF 10 kb)

Additional file 4: Neighbor-joining tree based on pair-wise simple matching dissimilarity index showing the genetic relationships among 101 individuals randomly re sampled from cultivated and wild korarima populations (19 populations were represented by five samples each and two populations were represented by three samples each). Blue color indicates individuals from cultivated populations whereas red represents individuals from wild populations. (DOCX 22 kb)

Additional file 5: UPGMA dendrogram based on pair wise simple matching dissimilarity index showing the genetic relationships among Ethiopian cultivated and wild korarima populations using SSR markers. The analysis resulted in two major clusters shown as “I” and “II”. (DOCX 27 kb)

Additional file 6: Allele frequency data for all korarima populations across the 11 SSR loci. (XLSX 18 kb)

## Abbreviations

AMOVA: Analysis of molecular variance
CTAB: Cetyl trimethyl ammonium bromide
EST: Expressed sequence tags
IBC: Institute of Biodiversity Conservation
NCBI: National Center for Biotechnology information
SNNP: Southern Nations, Nationalities, and Peoples’ Region
SSR: Simple sequence repeats
