# Supplementary material for: Genetic structure and relationships within and between cultivated and wild korarima [Aframomum corrorima (Braun) P.C.M. Jansen] in Ethiopia as revealed by simple sequence repeat (SSR) markers
Source: BMC Genet. 2017 Aug 1;18:72. doi: 10.1186/s12863-017-0540-4 (PMC5540420; doi:10.1186/s12863-017-0540-4)
Supplement: Supplementary file 1 — Passport data of A. corrorima samples collected from different sites in Ethiopia. (DOCX 17 kb) [file 12863_2017_540_MOESM1_ESM.docx]

| Zone | Specific location | Population name and code | No of samples | Latitude | Longitude | Altitude |
| --- | --- | --- | --- | --- | --- | --- |
| Illubabour^a^ | Sege Tageta | Gore_C1 (GC1-10) | 10 | 0890940 | 36 P 0777788 | 1924 |
|  | Adele Bise | Metu_C1 (MC1-10) | 10 | 0922384 | 36 P 0771124 | 1655 |
| Jimma^a^ | Sebeka Dibiya 1 | Jimma_C1 (JC1-10) | 10 | 0832213 | 37 N 0227907 | 1956 |
|  | Sebeka Dibiya 3 | Jimma_C2 (jc1-10) | 10 | 0833176 | 37 N 0229713 | 2045 |
|  | Sebeka Dibiya 2 | Jimma_C3 (JW1-10) | 10 | 0833173 | 37 N 0229730 | 2039 |
| Bench-Maji^a^ | Fanika-1 | Mizan-Teferi_C1 (mtc1-10) | 9 | 0768932 | 36 N 0776019 | 1336 |
|  | Fanika-2 | Mizan-Teferi_C2 (MTC1-10) | 10 | 0768822 | 36 N 0776059 | 1348 |
| Sheka^a^ | Beta Keble 1 | Masha_C1 (mac1-3) | 3 | 0856964 | 36 N 0773450 | 2221 |
|  | Beta Keble 2 | Masha_C2 (MaC1-10) | 10 | 0858605 | 36 N 0774026 | 2172 |
|  | Kubito-1 | Tepi_C1 (TC1-10) | 10 | 0809399 | 36 N 0763150 | 1885 |
|  | Kubito-2 | Tepi_C2 (tc1-10) | 10 | 0809737 | 36 N 0762409 | 1904 |
| Kefa^a^ | 01 kebele | Bonga_C1 (BC1-10) | 10 | 0806576 | 37 N 0193583 | 1657 |
|  | Beha | Bonga_C2 (BoC1-10) | 10 | 0803604 | 37 N 0195838 | 1701 |
|  | Around College | Bonga_C3 (bC1-10) | 10 | 0793460 | 37 N 0193220 | 1847 |
| Total^a^ |  |  | **132** |  |  |  |
| Illubabour^b^ | Adele Bise | Metu_W (mw1-3) | 3 | 0922920 | 36 P 0771391 | 1643 |
|  | Gumero | Gumero_W (GW1-10 | 10 | 0901233 | 36 P 0772847 | 1684 |
| Jimma^b^ | Sebeka Dibiya | Jimma_W (jw1-10) | 10 | 0832961 | 37 N 0229689 | 2057 |
|  | Afalo Naso | Jimma-Gera_W (jgw 1-10) | 10 | 0842462 | 37 N 0191504 | 1649 |
| Bench-Maji^b^ | Fanika | Mizan-Teferi_W (mtw1-10) | 10 | 0769786 | 36 N 0775205 | 1272 |
| Sheka^b^ | Betto Kebele | Masha_W (maw1-10) | 10 | 0860535 | 36 N 0775176 | 1841 |
| Kefa^b^ | Aermo Kebele | Bonga_W (bw1-10) | 10 | 0793119 | 37 N 0192835 | 1880 |
| Total^b^ | | | **63** |  |  |  |

^a^ = represent cultivated populations, ^b^ = represents wild populations, the format of latitude/longitude used were a Universal Transverse Mercator coordinate (UTM)
