# Supplementary material for: Genetic structure and relationships within and between cultivated and wild korarima [Aframomum corrorima (Braun) P.C.M. Jansen] in Ethiopia as revealed by simple sequence repeat (SSR) markers
Source: BMC Genet. 2017 Aug 1;18:72. doi: 10.1186/s12863-017-0540-4 (PMC5540420; doi:10.1186/s12863-017-0540-4)
Supplement: Supplementary file 2 — Characteristics of 23 microsatellite primers developed for Aframomum corrorima (The bold one are used for genetic diversity analysis) (DOCX 16 kb) [file 12863_2017_540_MOESM2_ESM.docx]

| Locus | Primer sequences (5 ′ –3 ′ ) | | Ta (°C) | Product size | GenBank accession no. |
| --- | --- | --- | --- | --- | --- |
| *Afco*_1 | F | TCTGAACCAGGGAATCCAAA | 64 | 157 | JX422070.1 |
|  | R | AACTCACTTGGAGGCCAACTT |  |  |  |
| *Afco*_2 | F | TTGACTTGGGTATGGCAAAA | 63 | 230 | JX422069.1 |
|  | R | AAGGTCGAGCAGGAGTAGCA |  |  |  |
| *Afco*_3 | F | GAATTCATGTTCTTGAGAAAAGTTTG | 63 | 198 | JX422068.1 |
|  | R | GCCAAATGAACGGACAGATT |  |  |  |
| *Afco*_4 | F | GCGAAACCCTCTCATCCTTA | 63 | 177 | JX422067.1 |
|  | R | CCATCTCCTCGTCCTTTTCA |  |  |  |
| *Afco*_5 | F | TGACTCCAAACTTGCAGGAG | 64 | 160 | JX422066.1 |
|  | R | AGCAGATCAATGCACGTGAG |  |  |  |
| *Afco*_6 | F | TCGACATGAAATCCCTACGAGA | 64 | 243 | JX422065.1 |
|  | R | GAGCTGTGAAGTGAAAGGGC |  |  |  |
| *Afco*_7 | F | AAAATTTCCAAACGGAAGATGA | 63 | 249 | JX422064.1 |
|  | R | TTGATTCTGCCTCCCATTTC |  |  |  |
| *Afco*_8 | F | GCTAACTTGTCTTTCCTATTTCTCC | 62 | 239 | JX422063.1 |
|  | R | TGGAAGCTGCATTCACTGAG |  |  |  |
| *Afco*_9 | F | TGTCCAAGGATTACTTATGAACGA | 62 | 202 | JX422062.1 |
|  | R | CGAATTGGAAAGTAATTATATGACC |  |  |  |
| *Afco*_10 | F | GCTATAGCCAAGGCAATTCG | 62 | 195 | JX422061.1 |
|  | R | TTCGATGTGTTTGCTAAGAAGA |  |  |  |
| *Afco*_11 | F | AATGCTTCTAGCTGGTTCGAC | 62 | 241 | JX422060.1 |
|  | R | CCTTGAATTTTATATTTCTTCCAGATG |  |  |  |
| *Afco*_12 | F | CAGATCGCAGTCGGATTCT | 63 | 247 | JX422059.1 |
|  | R | TCCGTTATTCACCGTTCGAT |  |  |  |
| *Afco*_13 | F | CTAGGGGATCGAACTGTGGA | 64 | 300 | JX422058.1 |
|  | R | CGCGTTTCTTCCCAATAAAA |  |  |  |
| *Afco*_14 | F | CCTTCCACGGTGTCTCATTT | 63 | 281 | JX422057.1 |
|  | R | TCATCCAAAACTTCAATCATGG |  |  |  |
| *Afco*_15 | F | ATCGATGGGATCGCCTTAC | 64 | 292 | JX422056.1 |
|  | R | GACGTCACGAATGTTGGTTG |  |  |  |
| *Afco*_16 | F | CTGCGTGCGAATTATCGAG | 64 | 393 | JX422055.1 |
|  | R | CTGAGGAGGACGAGAAGCTC |  |  |  |
| *Afco*_17 | F | TTTTTCATCGTTGTCCCTACG | 64 | 250 | JX422054.1 |
|  | R | GTCATCCAAAATGGCGACAC |  |  |  |
| *Afco*_18 | F | GGGAAAAGATTTCGACCTACA | 62 | 300 | JX422053.1 |
|  | R | GCACAAACTTCAGTAAGGATAATCA |  |  |  |
| *Afco*_19 | F | CAGACGAGAGGAGGGAGATG | 64 | 373 | JX422052.1 |
|  | R | CTCTGTGAGCCGTTCAATCC |  |  |  |
| *Afco*_20 | F | TCCACTGCGTATACCGCTAC | 65 | 245 | JX422051.1 |
|  | R | CTGGGCTATAGTGCCCATCG |  |  |  |
| *Afco*_21 | F | CGACAAGGAGGAGAAGAGGT | 63 | 250 | JX422050.1 |
|  | R | CCAACAGCCCTTCTTTTTGA |  |  |  |
| *Afco*_22 | F | GAAGAAGCGTTGGTGAGAGG | 64 | 468 | JX422049.1 |
|  | R | CTGTGTCGTCCAGCCGTATT |  |  |  |
| *Afco*_23 | F | AGAAGGATGTGCCCAAACC | 64 | 300 | JX422048.1 |
|  | R | CTCGCGATTTCAGGTCACTT |  |  |  |
