# Supplementary figures and images for: Genetic structure and relationships within and between cultivated and wild korarima [Aframomum corrorima (Braun) P.C.M. Jansen] in Ethiopia as revealed by simple sequence repeat (SSR) markers
Source: BMC Genet. 2017 Aug 1;18:72. doi: 10.1186/s12863-017-0540-4 (PMC5540420; doi:10.1186/s12863-017-0540-4)

$$\text{DeltaK} = \text{mean}(|L''(K)|) / \text{sd}(L(K))$$

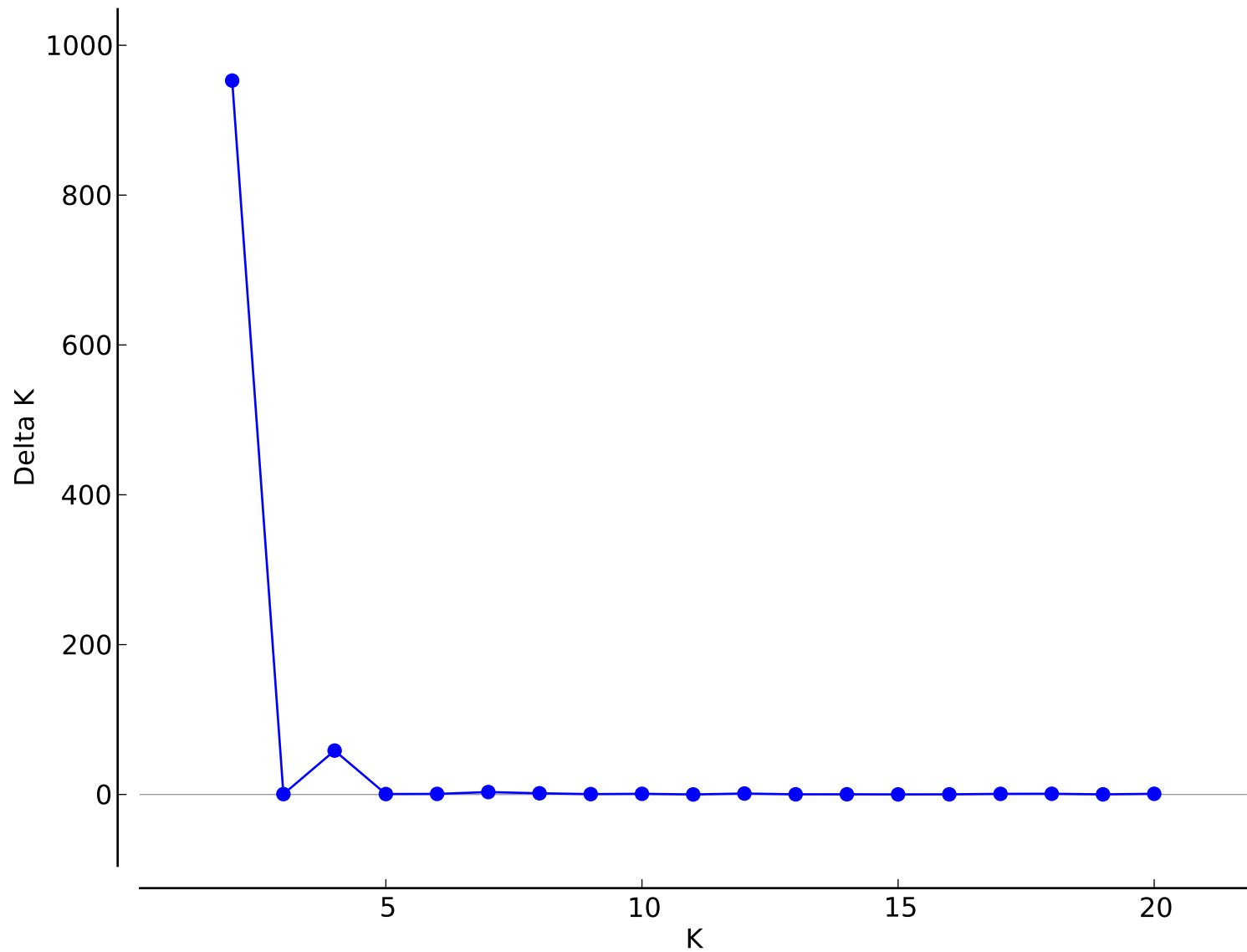

Supplement: Supplementary file 3 — A biplot detected the maximum peak at K = 2 (the optimum number of clusters) based on Evanno et al. 2005 [35] prediction. (PDF 10 kb) [file 12863_2017_540_MOESM3_ESM.pdf]

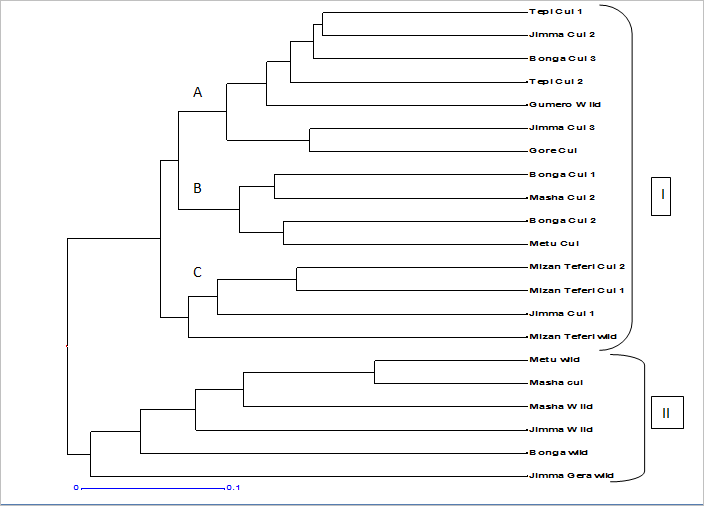

Supplement: Supplementary file 5 — UPGMA dendrogram based on pair wise simple matching dissimilarity index showing the genetic relationships among Ethiopian cultivated and wild korarima populations using SSR markers. The analysis resulted in two major clusters shown as “I” and “II”. (DOCX 27 kb) [file 12863_2017_540_MOESM5_ESM.docx]
