# Supplementary material for: Genetic structure and relationships within and between cultivated and wild korarima [Aframomum corrorima (Braun) P.C.M. Jansen] in Ethiopia as revealed by simple sequence repeat (SSR) markers
Source: BMC Genet. 2017 Aug 1;18:72. doi: 10.1186/s12863-017-0540-4 (PMC5540420; doi:10.1186/s12863-017-0540-4)
Supplement: Supplementary file 4 [file 12863_2017_540_MOESM4_ESM.docx]

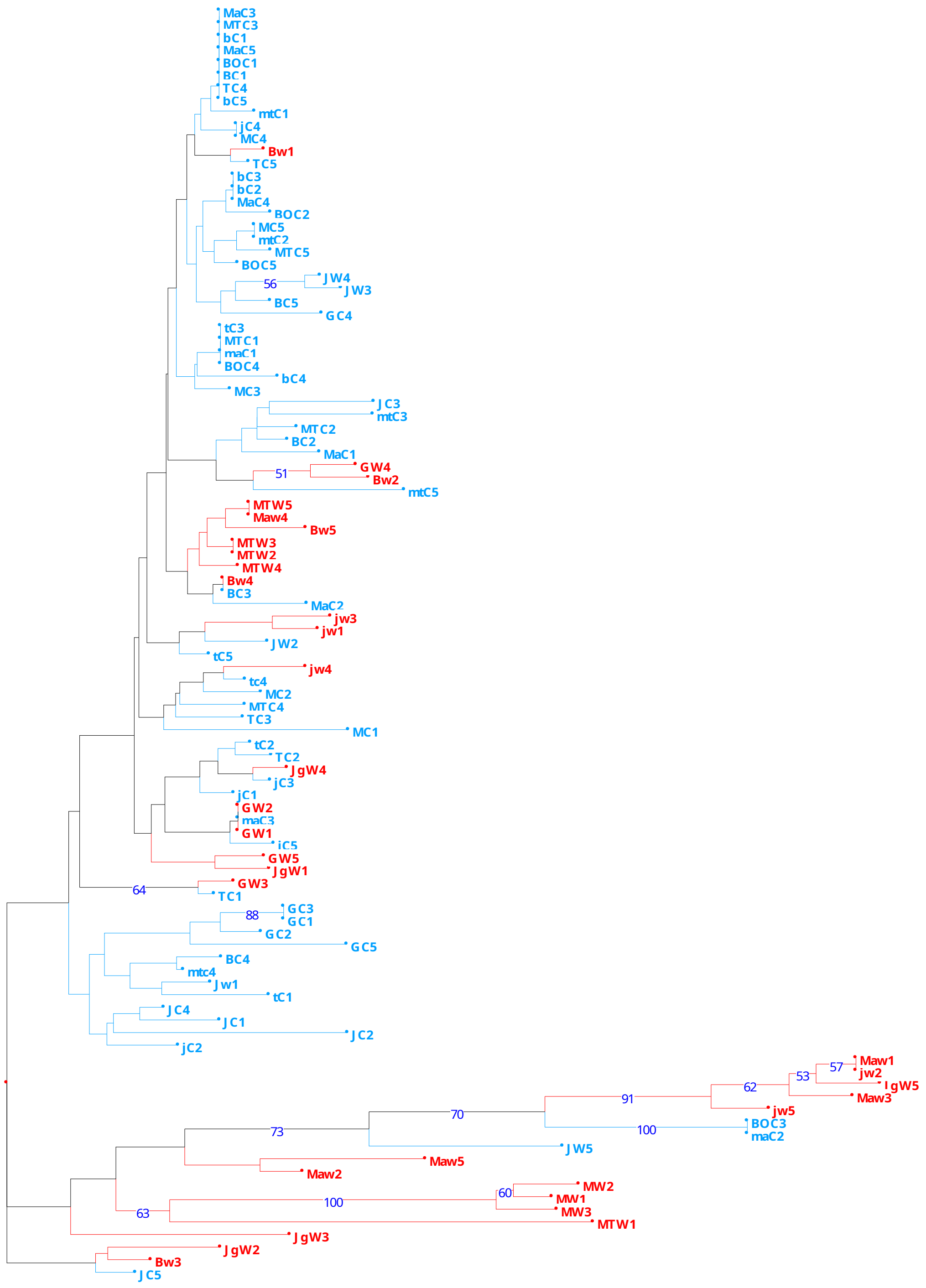
MaC3
MTC3
bC1
MaC5
BOC1
BC1
TC4
bC5
mtC1
jC4
MC4
Bw1
TC5
bC3
bC2
MaC4
BOC2
MC5
mtC2
MTC5
BOC5
JW4
56
JW3
BC5
GC4
tC3
MTC1
maC1
BOC4
bC4
MC3
JC3
mtC3
MTC2
BC2
MaC1
GW4
51
Bw2
mtC5
MTW5
Maw4
Bw5
MTW3
MTW2
MTW4
Bw4
BC3
MaC2
jw3
jw1
JW2
tC5
jw4
tc4
MC2
MTC4
TC3
MC1
tC2
TC2
JgW4
jC3
jC1
GW2
maC3
GW1
jC5
GW5
JgW1
GW3
64
TC1
GC3
88
GC1
GC2
GC5
BC4
mtc4
Jw1
tC1
JC4
JC1
JC2
jC2
Maw1
57
53
jw2
IgW5
62
Maw3
91
70
jw5
BOC3
100
maC2
73
JW5
Maw5
Maw2
MW2
60
MW1
100
MW3
63
MTW1
JgW3
JgW2
Bw3
JC5
